# Cav3.2 T-type calcium channels control acute itch in mice

**DOI:** 10.1186/s13041-020-00663-9

**Published:** 2020-09-01

**Authors:** Vinicius M. Gadotti, Joanna M. Kreitinger, Nicholas B. Wageling, Dylan Budke, Philippe Diaz, Gerald W. Zamponi

**Affiliations:** 1 Department of Physiology and Pharmacology Hotchkiss Brain Institute, Children’s Hospital Research Institute, Cumming School of Medicine, University of Calgary, Calgary, AB Canada; 2 Dermaxon LLC, Missoula, MT USA; 3 Department of Biomedical and Pharmaceutical Sciences, The University of Montana, Missoula, MT USA

**Keywords:** Itch, Pruritus, Histamine, Chloroquine, Cav3.2 T-type channel

## Abstract

Cav3.2 T-type calcium channels are important mediators of nociceptive signaling, but their roles in the transmission of itch remains poorly understood. Here we report a key involvement of these channels as key modulators of itch/pruritus-related behavior. We compared scratching behavior responses between wild type and Cav3.2 null mice in models of histamine- or chloroquine-induced itch. We also evaluated the effect of the T-type calcium channel blocker DX332 in male and female wild-type mice injected with either histamine or chloroquine. Cav3.2 null mice exhibited decreased scratching responses during both histamine- and chloroquine-induced acute itch. DX332 co-injected with the pruritogens inhibited scratching responses of male and female mice treated with either histamine or chloroquine. Altogether, our data provide strong evidence that Cav3.2 T-type channels exert an important role in modulating histamine-dependent and -independent itch transmission in the primary sensory afferent pathway, and highlight these channels as potential pharmacological targets to treat pruritus.

## Introduction

Acute itch (pruritus) serves a protective function in response to events such as insect bites, allergic reactions, or skin borne parasites [1, 2]. On the other hand, chronic itch reflects pathogenic, maladaptive, and debilitating conditions found in multiple systemic and skin diseases in pathologies such as diabetes, cancers, psoriasis and atopic dermatitis [3]. Akin to the detection of painful stimuli by nociceptors, itch related information is detected by pruriceptive sensory neurons that have their nerve endings in the skin, and is then transmitted to the brain via synaptic connections in the spinal cord and then through the spinothalamic tract [4–6]. Even though pruritus and pain are distinct sensations, they display anatomic similarities. Both are transmitted by unmyelinated C fibers and lightly myelinated Aδ fibers [6]. These smaller diameter neurons express voltage gated calcium channels (VGCC) including members of the T-type calcium channel family that are essential for pain signaling [7]. The latter belong to the family of the low voltage activated calcium channels [8] and are further subdivided into Cav3.1, Cav3.2, and Cav3.3 subtypes, with expression in both the central and peripheral nervous systems. The Cav3.2 subtype is prominently expressed in somatosensory afferent fibers and spinal cord neurons and is a fundamental regulator of sensory signalling to the spinal cord [9, 10]. While the involvement of Cav3.2 in the transmission of pain is clearly established, the roles of these channels in the transmission of itch is only emerging. Indeed, it was reported that Cav3.2 is important for hydrogen sulfide (H_2_S)-induced itch responses in mice [11]. More recently, it was also demonstrated that Cav3.2 channels are upregulated in the skin of uremic itch sufferers and may thus contribute to itch transmission in these patients [12]. Altogether, these findings suggest that T-type calcium channels may also be important for the activity of pruriceptive neurons, thus we sought to determine whether Cav3.2 calcium channels are involved in chloroquine- and histamine-induced itch. We show that itch responses are reduced in Cav3.2 knockout mice and by pharmacological intervention with the T-type calcium channel blocker DX332 [13].

## Materials and methods

### Animals

All experiments were performed following approval by the Animal care committee of the University of Calgary. Adult (7–10 weeks old) male or female C57BL/6 J wild-type or male CACNA1H knockout (Cav3.2 null) mice were used and purchased from Jackson laboratories. Mice were housed at a maximum of five per cage (30 × 20 × 15 cm) with free access to food and water. They were kept on a 12-h light/dark cycle (lights on at 7 am) with the room maintained at a temperature of 23 ± 1 °C. Experiments were carried out between 9 am-3 pm. For each condition, separate randomized groups of mice were tested on three different days. Drugs were delivered subcutaneously (s.c.) and standard volumes of 20 μl were injected. DX332 was dissolved in dimethyl sulfoxide (DMSO), and control animals received phosphate buffered solution (PBS) + 1% DMSO, which was the maximum DMSO concentration in the highest dosage of DX332 tested. Different cohorts of mice were used for each test and each mouse was used only once. Chloroquine diphosphate (Tocris) and histamine (Sigma-Aldrich) were dissolved in PBS. The selective Cav3.2 blocker DX332 [13] was dissolved in DMSO 2% and was co-injected subcutaneously (s.c.) with the pruritogens.

### Histamine-induced itch

Histamine (100 μg in 20 μl) or PBS were injected subcutaneously at the back of the neck of wild-type or Ca_V_3.2 null mice using a BD insulin syringe with a 31-gauge needle. A volume of 20 μl was injected. Animals had the fur on their back trimmed 48 h before the experiments. After receiving an injection of a solution of histamine or PBS, mice were placed into individual plexiglass holding containers and the time spent scratching was scored for 30 min. In a different series of experiments, either male or female wild-type mice were treated with DX332 (100 μg/s.c.) or vehicle (20 μl/s.c.) in association with histamine.

### Chloroquine-induced itch

In the same manner as above, injections of 20 μl of chloroquine (200 μg) were performed using a BD insulin syringe with a 31-gauge needle. Injections occurred in the back of the neck 2 days after having the fur trimmed. Following an injection with chloroquine or PBS, animals were placed and kept in plexiglass holding containers and the time spent scratching was scored for 30 min. Male and female wild-type mice were treated with DX332 (10.0–100.0 μg/s.c.) or vehicle (20 μl/s.c.) in association with chloroquine.

### Statistical analysis

Data were analyzed with Graphpad Prism 6.0 and are presented as the mean ± SEM. One- or two-way analysis of variance (ANOVA) with Tukey’s post hoc correction was used. Statistical significance was accepted at the level of *p* < 0.05.

## Results and discussion

**Fig. 1:**
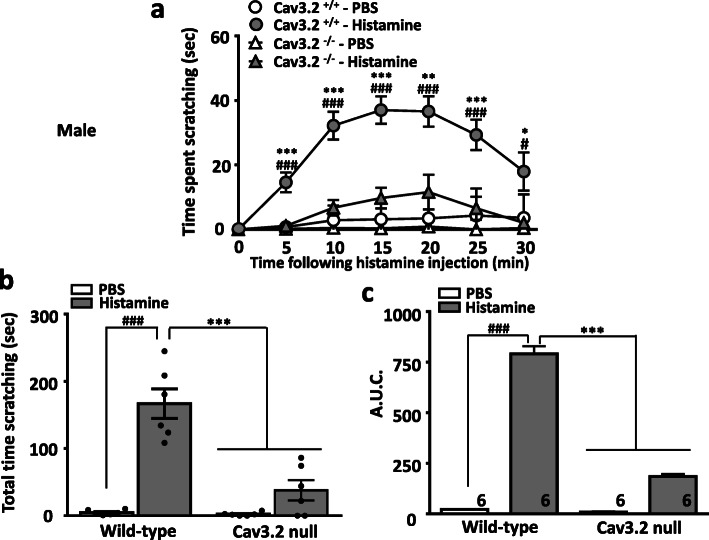
Histamine elicits scratching behavior in wild type but not in Cav3.2 null mice. **a** Time course of scratching behaviors, **b** total time scratching, and **c** area under the curve of the effect of a single subcutaneous injection of histamine (100 μg/20 μl) either wild type or Cav3.2 null mice. Each symbol/bar represents the mean ± S.E.M. Numbers reflect mice tested. Two-way ANOVA reveals behavioural abnormalities ^#^ *P* < 0.05, ^###^ *P* < 0.001 PBS vs histamine and * *P* < 0.05, ** *P* < 0.01 and *** *P* < 0.001 WT vs. Cav3.2 null mice

**Fig. 2:**
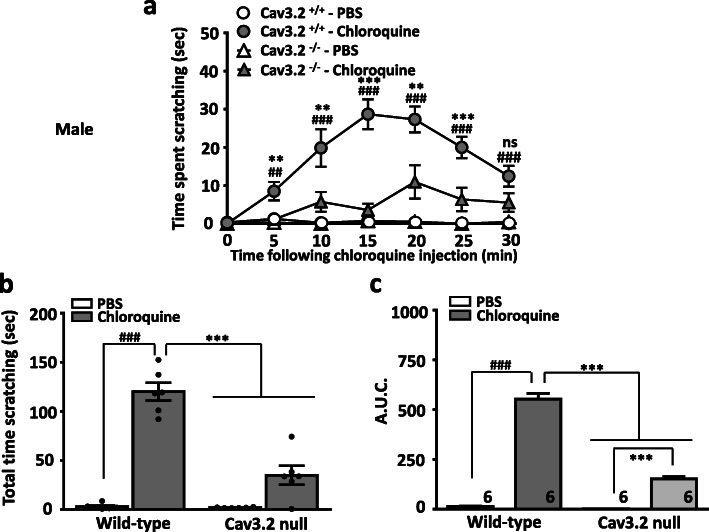
Chloroquine evokes scratching behavior in wild type but not in Cav3.2 null mice. **a** Time course of scratching behaviors, **b** total time scratching, and **c** area under the curve of the effect of a single subcutaneous injection of chloroquine (200 μg/20 μl) either wild type or Cav3.2 null mice. Each symbol/bar represents the mean ± S.E.M. Numbers reflect mice tested. Two-way ANOVA reveals behavioural abnormalities ^# #^*P* < 0.01, ^###^ *P* < 0.001 PBS vs chloroquine and ** *P* < 0.01, *** *P* < 0.001 WT vs. Cav3.2 null mice or PBS vs histamine in Cav3.2 null mice

We first determined whether deletion of Cav3.2 channels in mice affects scratching behavior induced by histamine or chloroquine injection. Subcutaneously injected histamine (100 μg in 20 μl) into the nape of the neck of wild-type mice elicited robust scratching behavior (Fig. 1), in agreement with previous works [14]. In contrast, Cav3.2 null mice were resistant to histamine induced itch as seen by a dramatic reduction in the time spent scratching (Fig. 1). In a similar manner, injections of chloroquine (200 μg in 20 μl) into the nape of the neck of wild-type mice produced strong and sustained scratching responses that were greatly depressed in Cav3.2 null mice (Fig. 2). Together, these data indicate that the expression of Cav3.2 channels is required for acute itch.

**Fig. 3:**
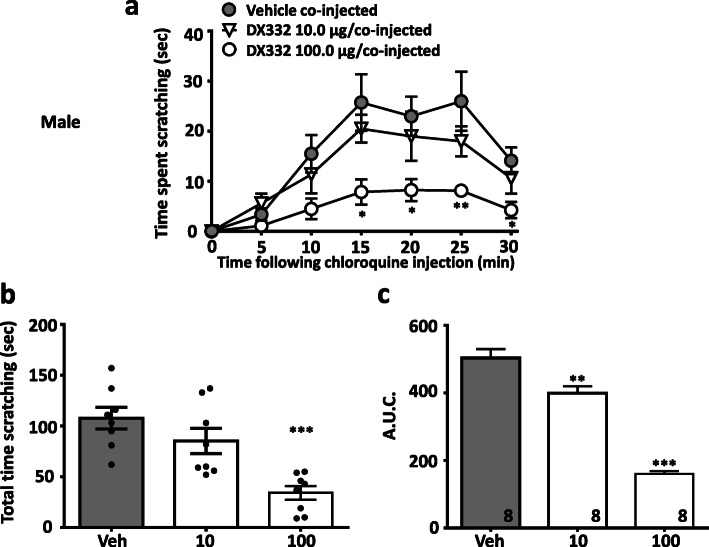
DX332 reduces acute itch-related behavior induced by chloroquine in male mice. **a** Time- and dose- dependence, **b** total time scratching, and **c** area under the curve of the of anti-pruritogenic activity caused by DX332 (10.0–100.0 μg/co-injected). Each symbol/bar represents the mean ± S.E.M. Numbers reflect mice tested. Two-way ANOVA reveals drug induced inhibition of behavioural abnormalities * *P* < 0.05, ** *P* < 0.01, *** *P* < 0.001

**Fig. 4:**
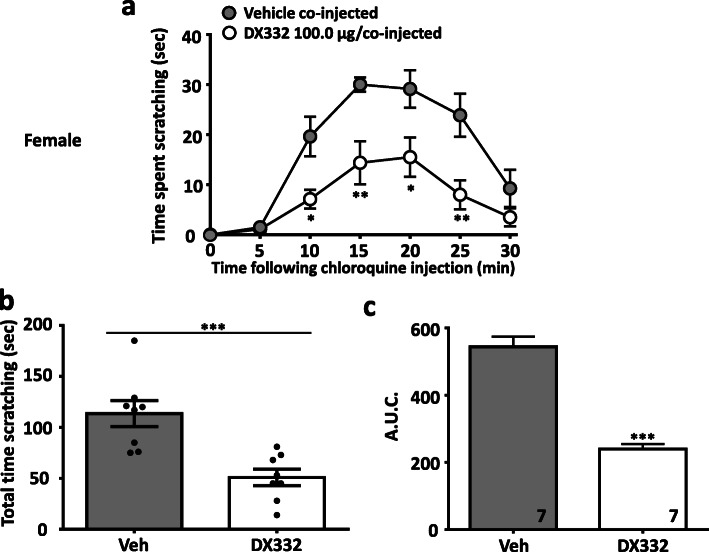
DX332 reduces acute itch-related behavior induced by chloroquine in female mice. **a** Time-dependence, **b** total time scratching, and **c** area under the curve of the of anti-pruritogenic activity caused by DX332 (100.0 μg/co-injected). Each circle/bar represents the mean ± S.E.M. Numbers reflect mice tested. Two-way ANOVA reveals drug induced inhibition of behavioural abnormalities * *P* < 0.05, ** *P* < 0.01, *** *P* < 0.001

**Fig. 5:**
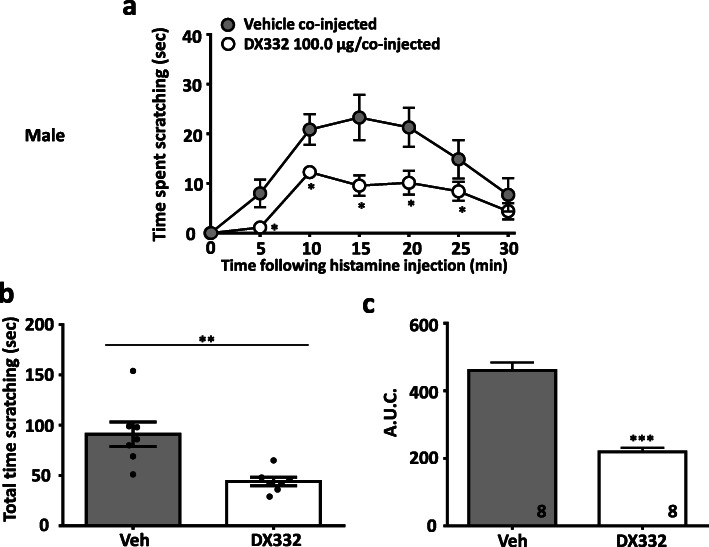
*DX332 reduces acute itch-related behavior induced by histamine in male mice. (***a)** Time-dependence, **(b)** total time scratching, and **(c)** area under the curve of the of anti-pruritogenic effect caused by DX332 (100.0 μg/co-injected). Each circle/bar represents the mean ± S.E.M. Numbers reflect mice tested. Two-way ANOVA reveals drug induced inhibition of behavioural abnormalities * *P* < 0.05, ** *P* < 0.01, *** *P* < 0.001

**Fig. 6:**
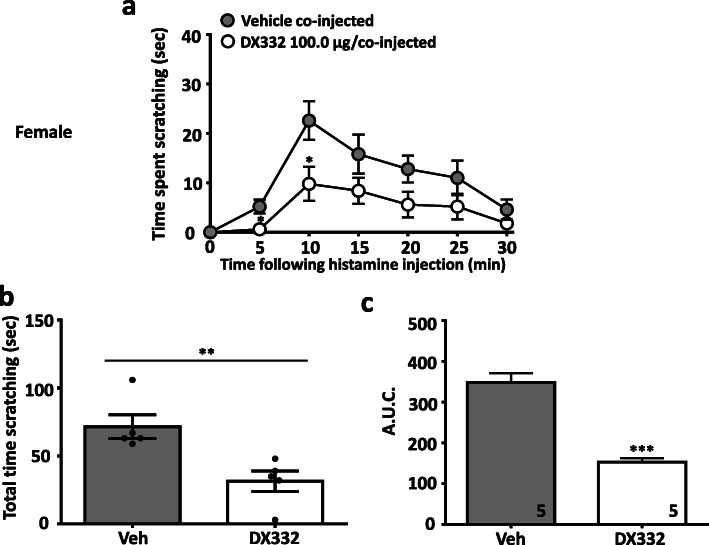
DX332 reduces acute itch-related behavior induced by histamine in female mice. **a** Time dependence, **b** total time scratching, and **c** area under the curve of the of anti-pruritogenic effect caused by DX332 (100.0 μg/co-injected). Each circle/bar represents the mean ± S.E.M. Numbers reflect mice tested. Two-way ANOVA reveals drug induced inhibition of behavioural abnormalities * *P* < 0.05, ** *P* < 0.01, *** *P* < 0.001

In a separate series of experiments, we evaluated the effect of the T-type channel blocker DX332 (a.k.a. compound 9 in Ref [13]) in male and female wild-type mice. DX332 is a potent inhibitor of Cav3.2 channels and has been shown by us to mediate analgesia in mouse models of inflammatory and neuropathic pain in a Cav3.2 channel-dependent manner [13]. Treatment with DX332 (10.0–100.0 μg in 20 μl, co-injected) produced dose-dependent inhibition of itch responses in male (Fig. 3) mice injected with chloroquine. A similar effect was observed in female mice (Fig. 4). Again, DX332 (100.0 μg in 20 μl, co-injected) produced a significant decrease of histamine–induced scratching behaviors in male (Fig. 5) and in female (Fig. 6) mice. We did not observe any flinching or vocalization in mice injected with either chloroquine or histamine, thus indicating an absence of behaviors that are a typical indication of nociceptive or nocifensive responses rather than itch [15]. Collectively, our data indicate that local inhibition of Cav3.2 channels in tissues exposed to histamine or chloroquine prevents the development of itch. Previous work suggested that Cav3.1 and Cav3.3 channels, but not Cav3.2, are determinants for itch signal transmission to the spinal cord [16], however, these experiments were based on intradermal injection of zinc which (although being a preferential inhibitor of Cav3.2 among the T-type calcium channel family) also enhances the activity of Cav3.1 and Cav3.3 channels by slowing inactivation kinetics [17]. By contrast, our use of Cav3.2 null mice allows us to clearly attribute a role of Cav3.2 in the processing of peripheral itch signals.

Itch is transmitted by diverse afferent sensory neurons located in the dorsal root ganglia (DRG) and trigeminal ganglia (TG) which detect nociceptive and pruritogenic stimuli and can be modulated by pruritogens as well as algogens [1, 18, 19]. Despite the neurophysiological similarities between pain and itch, there is accumulating evidence that itch involves unique cellular and molecular mechanisms as well as distinct peripheral and central neuronal circuitry [20]. There are two well characterized types of chemical itch, one known as histamine-dependent and another as histamine-independent itch, both of which are mediated by unmyelinated C fibers and lightly myelinated Aδ fibers [21]. In fact, while some lines of evidence argue that pathways for histamine- and non histamine-mediated itch may almost completely overlap, in more recent studies others have suggested that distinct neuronal pathways mediate histamine and non-histamine itch [14, 22, 23]. Our data suggest that irrespective of these details, Cav3.2 channels are a common feature to both histamine-dependent and histamine-independent itch.

Histamine and chloroquine activate distinct cellular signalling pathways to give rise to itch [24]. This is underscored by the notion that histamine receptor antagonists are effective in histamine mediated pruritus [25] and allergenic itch, whereas they are ineffective against most types of chronic itch conditions such as eczema and dry skin itch [17, 26, 27]. Histamine-mediated itch is initiated by activation of the histamine type 1 receptor (H1) expressed in TRPV1^+^/phospholiphase-β-3 (PLCB3) positive neurons [14], and requires co-activation of the transient receptor potential cation channel subfamily V member 1 (TRPV1) and H1 receptors to elicit behavioral responses [28]. On the other hand, itch produced by chloroquine is mediated by the Mas-related G-protein-coupled receptor (Mrgpr) A3 or C1, and with co-activation of Transient receptor potential cation channel subfamily A member 1(TRPA1) channels [23, 29]. Both groups are members of the GPCR superfamily and are coupled to various G-proteins, through which they transduce their signals via second messengers such as phospholipase Cbeta 3 (PLCB3) and inositol triphosphate (IP3) [14]. It is possible that Cav3.2 channels are direct downstream targets of these signalling pathways, thus leading to increased T-type channel activity and increased neuronal firing. Alternatively, it is conceivable that histamine may directly act on Cav3.2 channels. Finally, it is possible that the activity of pruriceptive sensory neurons is increased by receptor-mediated actions on other types of ion channels, and that pharmacological inhibition or depletion of Cav3.2 channels expressed in nerve endings simply serves to compensate for this increase in activity. At this point we cannot distinguish among the alternatives. The Cav3.2 channel is widely expressed in TRPV1 positive sensory fibers where it is known to participate in the transmission of pain signals from the nerve endings to the spinal cord [30]. It was demonstrated that mechanical hyperalgesia and allodynia caused by hydrogen sulfide (H_2_S) require activation of both Cav3.2 channels and TRPA1 receptors [31]. Of interest, TRPV1- and TRPA1- positive fibers participate in different modalities of pain, including inflammatory mechanical hyperalgesia, mechanical allodynia and visceral pain [32], in which Cav3.2 channels have also emerged as potential therapeutic targets [33, 34]. The Cav3.2 channel is also considered as a selective marker for low (C-) threshold mechanoreceptors (LTMRs) that express Tyrosine hydroxylase/VGLUT3/TAFA4 and for the medium (Aδ-) LTMRs that are positive for TrkB [7]. The precise expression and distribution of Cav3.2 channels in pruriceptive neurons is not known, however, given that itch responses were inhibited by local administration of DX332, they must be functionally involved at the level of nerve endings.

In conclusion, our findings reveal that the Cav3.2 T-type channel subtype is involved in both histamine-dependent and histamine-independent acute itch responses in mice of both sexes. While additional studies are necessary to elucidate the role played by the Cav3.2 channel in chronic itch such as atopic dermatitis, our findings strongly demonstrated that targeting Cav3.2 channels could be exploited for the development of novel anti-pruritus therapies.

## Data Availability

The data used in our study are available from the authors on reasonable request.

## Abbreviations

LTMR: Low threshold mechanoreceptor
VGCC: Voltage gated calcium channel
TRPV1: Transient receptor potential cation channel subfamily V member 1
H1: Histamine type 1 receptor
PLCB3: Phospholiphase-β-3
DMSO: Dimethyl sulfoxide
PBS: Phosphate buffered saline
